# Inhibition of apoptosis prevents West Nile virus induced cell death

**DOI:** 10.1186/1471-2180-7-49

**Published:** 2007-05-29

**Authors:** Malte C Kleinschmidt, Martin Michaelis, Henry Ogbomo, Hans-Wilhelm Doerr, Jindrich Cinatl

**Affiliations:** 1 Institute for Medical Virology, Johann Wolfgang Goethe-University, Paul-Ehrlich-Str. 40, 60596 Frankfurt am Main, Germany

## Abstract

**Background:**

West Nile virus (WNV) infection can cause severe meningitis and encephalitis in humans. Apoptosis was recently shown to contribute to the pathogenesis of WNV encephalitis. Here, we used WNV-infected glioma cells to study WNV-replication and WNV-induced apoptosis in human brain-derived cells.

**Results:**

T98G cells are highly permissive for lytic WNV-infection as demonstrated by the production of infectious virus titre and the development of a characteristic cytopathic effect. WNV replication decreased cell viability and induced apoptosis as indicated by the activation of the effector caspase-3, the initiator caspases-8 and -9, poly(ADP-ribose)polymerase (PARP) cleavage and the release of cytochrome c from the mitochondria. Truncation of BID indicated cross-talk between the extrinsic and intrinsic apoptotic pathways. Inhibition of the caspases-8 or -9 inhibited PARP cleavage, demonstrating that both caspases are involved in WNV-induced apoptosis. Pan-caspase inhibition prevented WNV-induced apoptosis without affecting virus replication.

**Conclusion:**

We found that WNV infection induces cell death in the brain-derived tumour cell line T98G by apoptosis under involvement of constituents of the extrinsic as well as the intrinsic apoptotic pathways. Our results illuminate the molecular mechanism of WNV-induced neural cell death.

## Background

West Nile Virus (WNV), a single stranded RNA flavivirus, is a member of the Japanese encephalitis virus (JEV) serocomplex. Initially isolated in 1937, it has become endemic in Africa, the Middle East and parts of Asia and Europe [1,2]. WNV infection of humans typically results in subclinical or non-specific, mild febrile illnesses. However, about 1 in 150 patients will develop encephalitis and meningitis with high lethality rate due to virus invasion into the central nervous system (CNS) [3]. Since 1999, the virus has increasingly gained importance in North America as it caused an epizootic among birds and horses and an epidemic of meningitis and encephalitis in humans [3]. To date, no pharmacological treatment options for WNV-infected patients exist.

Apoptosis is a highly conserved mode of programmed cell death, commonly mediated by the activation of caspases [4]. Although neurons are regarded as the major target of WNV in vivo [1], WNV infection has been shown to induce apoptosis in different cell lines in a similar manner in vitro [5-8]. This includes a wide range of different cell types such as mouse fibroblasts (NIH/3T3), mouse neuroblastoma cells (Neuro-2A), mouse embryonic stem cells, Vero cells (African green monkey kidney cell line), human ovarian carcinoma cells (HeLa), human neuroblastoma cells (SH-SY5Y), leukemic cells (K562), and human rhabdomyosarcoma cells (RD) [5-9]. Different WNV proteins (i.e. envelope 1 (E) and non-structural protein 3 (NS3)) were shown to induce caspase-dependent apoptosis when transfected into cells [7,8]. Analysis of the influence of WNV infection on the gene expression of A172 (human glioma) cells using microarray technique indicated upregulation of apoptosis-related genes [10]. Although neural cells were used, no further evaluation of the distinct mechanism of cell death was performed. Recently, it was shown that WNV-infection induces caspase-3 activation and apoptosis in brains of wild-type mice and in primary CNS-derived mouse neurons [11]. Both, caspase-3^-/- ^genotype as well as treatment with caspase inhibitors decreased virus-induced cell death. However, role of upstream apoptosis pathways was not studied.

WNV infection-induced cell death may contribute to fatal WNV disease [11,12]. Therefore, thorough knowledge of the molecular mechanism of WNV-induced neural cell death will allow us to better understand the progression of WNV infection and the associated neurological pathology. Here, we used human glioma cell line T98G that is highly susceptible to WNV-induced apoptosis to investigate the contribution of caspase-dependent apoptosis to WNV infection-induced cell death. The potential of caspase inhibitors to increase viability of WNV-infected cell cultures was examined.

## Results

### Virus growth

**Figure 1:**
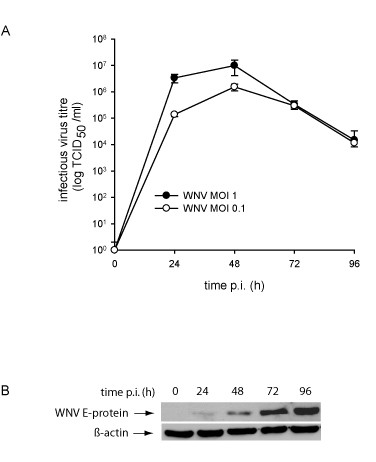
T98G cells are permissive for WNV infection. Production of infectious virus titres (A) and accumulation of viral envelope antigen (B) in T98G cell cultures infected with WNV at different time points p.i. Values represent the mean (± SD) of three different experiments. The blot shown is representative for a set of three different experiments.

When T98G cell cultures were infected at MOI 0.1 infectious virus titres increased from undetectable levels at 1 h p.i. to a maximum of 1.54 × 10^6 ^TCID_50_/ml at 48 h p.i. (Fig. 1a). Infection at MOI 1 resulted in 24.5-fold (3.36 × 10^6 ^vs. 1.37 × 10^5^) and 6.4-fold (9.92 × 10^6 ^vs. 1.54 × 10^6^) higher infectious titres 24 and 48 h p.i. relative to cultures infected at MOI 0.1. Both infectious virus titres of cultures infected at MOI 0.1 and MOI 1 rapidly decreased after reaching their maximum and were similar at 72 h (2.97 × 10^5 ^vs. 3.36 × 10^5^) and 96 h (1.15 × 10^4 ^vs. 1.49 × 10^4^) p.i. In contrast to decreasing infectious virus titres after a maximum at 48 h p.i., intracellular virus E protein accumulated in the infected cells as shown by Western Blot analysis (Fig. 1b).

### Induction of cytopathic effect (CPE)

**Figure 2:**
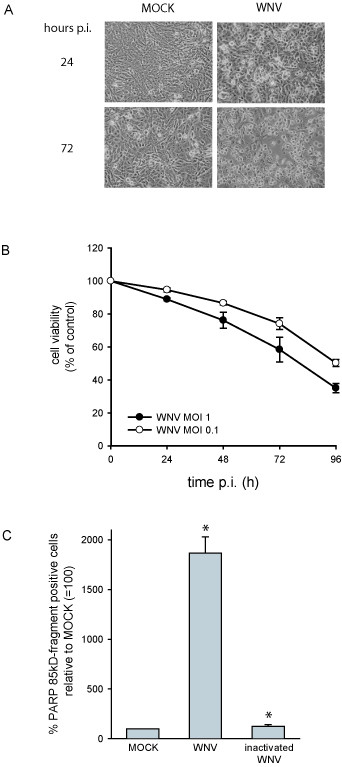
Effects of WNV on T98G cell viability. WNV-induced CPE (MOI 1) (A), cell viability of WNV-infected T98G cell cultures relative to mock-infected T98G cells (B) and virus induced PARP cleavage (MOI 1) (C) in infected cells. Values represent the mean (± SD) of three different experiments and pictures shown are representative for a set of three different experiments. *p < 0.05 compared to mock-infected cultures.

In T98G cells infected at MOI 1, first signs of a characteristic CPE appeared 24 h p.i. impressing as rounding of the cells and detachment from the monolayer. The intensity of CPE increased with time and was highly noticeable at 72 h p.i. (Fig. 2a). In cultures infected at MOI 0.1 occurrence of CPE was delayed by approximately 24 h relative to cultures infected at MOI 1 (data not shown).

### WNV infection reduces cell viability and induces PARP cleavage in T98G cells

WNV infection markedly decreased cell viability of T98G cells in a time dependent manner as measured by MTT assay (Fig. 2b). This loss of viability was also dependent on the MOI at which the cultures were infected. At 96 h p.i. cell viability of WNV-infected cultures decreased to 34.9 and 50.2% when infected at MOI 1 and 0.1 respectively. To show whether WNV-induced cell death was due to apoptosis quantitative analysis of PARP cleavage into proteolytic fragments was performed. At 48 h p.i. the amount of cells that stained for the 85 kD fragment of PARP was 18.17-fold higher than that of the mock infected control. Induction of apoptosis depended on viral replication as inactivated WNV failed to induce PARP cleavage (Fig. 2c).

### WNV infection induces caspase activation and cytochrome c release in T98G cells

**Figure 3:**
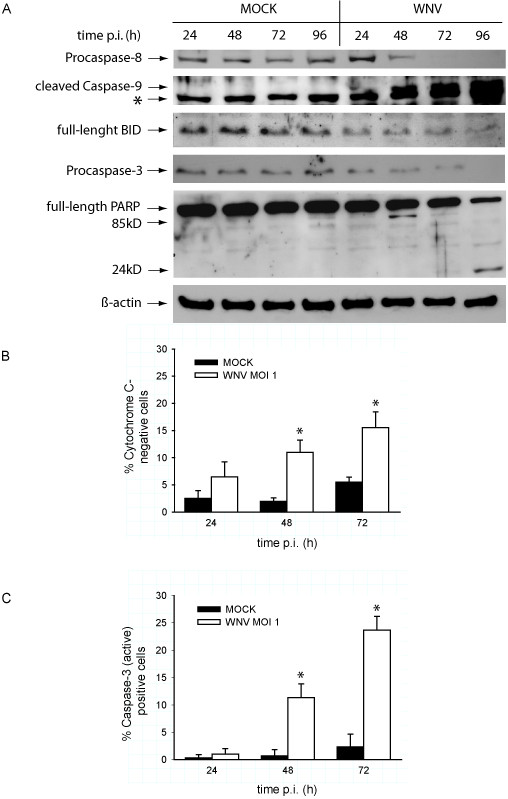
WNV infection induces caspase activation and cytochrome c release. Cellular levels of procaspase-8, cleaved caspase-9, BID and procaspase-3 during WNV-infection (A), release of cytochrome c from mitochondria into the cytoplasma (B) and caspase-3 activation (C) in WNV infected cells (all MOI 1). The band in A marked with * resulted from unspecific antibody binding. Blots shown are representative for a set of three different experiments. Values represent mean (± SD) from three independent experiments. *p < 0.05 compared to mock-infected cultures.

Western blots performed with protein extracts of WNV-infected and mock-infected cultures at 24, 48, 72 and 96 h p.i. showed cleavage of the initiator caspases, caspase-9 and caspase-8 and truncation of BID in infected cells. A 54 kD band, representing full-length pro-caspase-8, was detected in all the lysates of mock-infected cultures with constant intensity. In WNV-infected cultures (MOI 1) this band decreased in intensity and was below detectable level at 96 h p.i. (Fig. 3a). A 37 kD band, corresponding to the cleavage product of caspase-9, was absent in the protein lysates of mock-infected cultures but became apparent in the protein lysates of cultures infected with WNV (MOI 1) at 48, 72 and 96 h p.i. The intensity of this 37 kD band increased with time (Fig. 3a). In all mock-infected cultures a 22 kD band representing full-length BID was detected with constant intensity. In WNV-infected cultures this band intensity decreased, indicating BID truncation during WNV-infection (Fig. 3a).

Quantification of cytochrome c release showed that at 24 h p.i. 6.5% of the WNV infected cells (MOI 1) did not stain for mitochondrial cytochrome c compared to 2.5% of the mock infected control. The amount of negative cells increased to 10.99 and 15.52% at 48 and 72 h p.i., respectively in WNV-infected cells compared to 1.96 and 5.51% of the mock infected control (Fig. 3b). Activation of caspase-3 was additionally quantified by immune-staining of infected cells. At 48 h p.i. 11.3% of the cells infected with WNV (MOI 1) stained for cleaved caspase-3 compared to 1.2% of the mock infected control. This amount increased to 23.7% at 72 h p.i. compared to 2.3% of the mock infected control (Fig. 3c).

### Inhibition of WNV-induced PARP-cleavage by caspase inhibitor peptides

**Figure 4:**
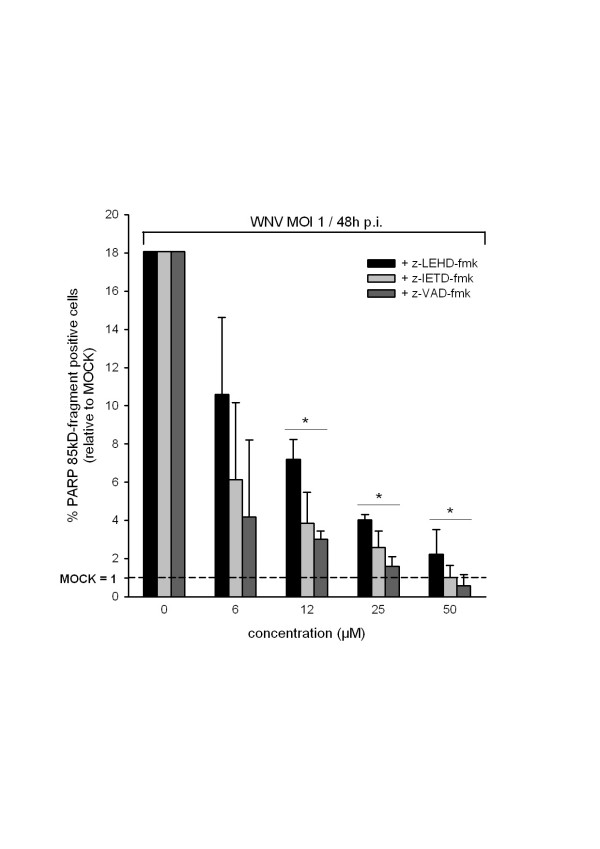
Caspase inhibitor peptides inhibit WNV-induced PARP-cleavage. Effect on WNV-induced PARP cleavage of peptides z-IETD-fmk (caspase-8 inhibitor), z-LEHD-fmk (caspase-9 inhibitor) and z-VAD-fmk (pancaspase inhibitor) at 48 h p.i. Results are expressed as percentage of values obtained from WNV-infected cultures (MOI 1) without any inhibitor added. Values represent mean (± SD) from three independent experiments. *p < 0.05 compared to untreated virus control.

Virus-induced PARP cleavage was strongly inhibited when caspase-8 inhibitor (z-IETD-fmk) caspase-9 inhibitor (z-LEHD-fmk) and pan-caspase inhibitor (z-VAD-fmk) were added directly to the cell culture medium of WNV-infected cells (Fig. 4). All caspase inhibitors inhibited PARP cleavage in a dose-dependent manner. Z-VAD-fmk exerted the strongest inhibitory effects on PARP cleavage, followed by z-IETD-fmk. However, addition of z-LEHD-fmk resulted in the weakest inhibition of PARP cleavage.

### Pan-caspase inhibition prevents WNV-induced cell death without affecting infectious virus titre production

**Figure 5:**
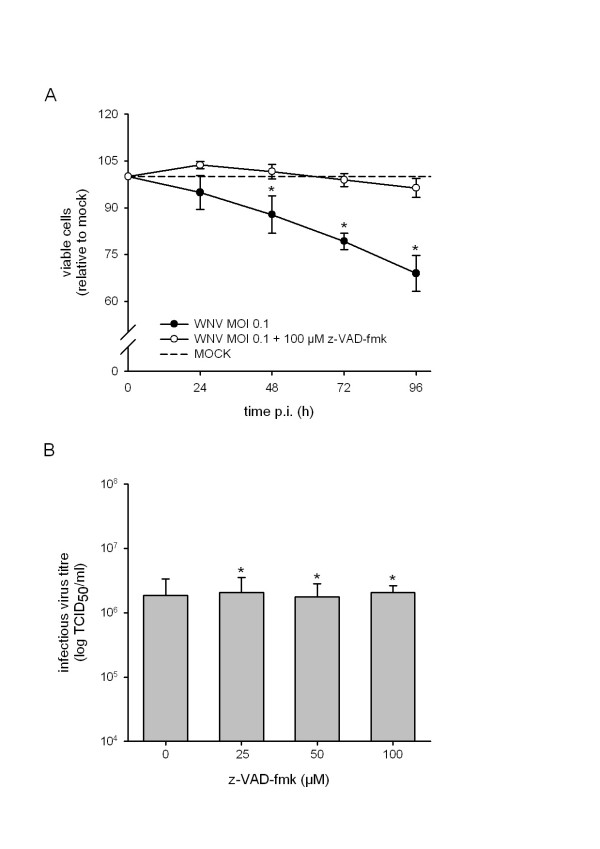
Prevention of WNV-induced cell death by pan-caspase inhibition. Inhibition of virus-induced cell death indicated by trypan blue staining of WNV (MOI 0.1)-infected T98G cell cultures by pan-caspase inhibitor z-VAD-fmk (100 μM) relative to mock-infected cells (A) and the effect of different z-VAD-fmk concentrations on infectious virus titre production (TCID_50_/ml, 48 h p.i.) after infection at MOI 0.1 (B). Values represent mean (± SD) from three independent experiments. *p < 0.05 compared to untreated virus control.

The addition of 100 μM z-VAD-fmk directly to the cell culture medium of WNV-infected (MOI 0.1) T98G cell cultures prevented virus-induced cell killing as assessed by the trypan blue dye exclusion assay (Fig. 5a). To exclude that this finding was an effect of altered viral replication due to z-VAD-fmk treatment, virus-titres of WNV-infected cultures (MOI 0.1) treated with 0, 25, 50 and 100 μM z-VAD-fmk were compared. Caspase inhibition had no effect on infectious virus titres at 48 h p.i. (Fig. 5b).

## Discussion

The human glioma cell line T98G is permissive for WNV infection as demonstrated by the production of infectious virus and viral antigens. In the present study, we used this cell culture system to study effects of WNV infection on apoptosis induction in brain-derived cells. WNV infection killed T98G cells in a time dependent manner. Although maximum virus titre was achieved 48 h p.i., WNV proteins continued to accumulate in infected cells at later times. The accumulation of viral proteins correlated with decreasing cell viability and the level of observed cytopathic effects. Similar observation demonstrating that the induction of cell death can happen independently of WNV-replication were made before using other cell types including human kidney cell line 293 and human retinal pigment epithelial cells [17,18]. Taken together, these data suggest that the treatment with inhibitors of virus replication at later time points p.i. may be limited in preventing WNV-induced pathogenicity. Therefore, substances that inhibit WNV-induced cytotoxic effects may be used as additional treatment options in the therapy of WNV diseases to improve outcome.

Many viruses including WNV have been shown to induce apoptosis in their target cells in vitro and in vivo [6,19-21]. Activation of both the intrinsic and extrinsic apoptotic pathways has been described during viral infections. The extrinsic apoptotic pathway is initiated by binding of death activators (e.g. Fas-ligand) to their respective transmembrane death receptors. This binding allows interaction with cytoplasmatic adaptor proteins (e.g. Fas associated death domain) which subsequently activates caspase-8. The activation of caspase-8 leads to effector caspases (e.g. caspase-3 or -7) activation and the degradation phase of apoptosis [22]. The intrinsic apoptotic pathway is initiated by signalling from pro-apoptotic proteins of the Bcl-2 family such as Bax which trigger mitochondria-outer-membrane-permeabilisation (MOMP) followed by the release of cytochrome c from mitochondria into the cytoplasma in the induction phase. Cytochrome c then associates with Apaf-1, caspase-9, and ATP to form the apoptosome. The apoptosome, similar to caspase-8 then induces the degradation phase of apoptosis via activation of effector caspases [4]. These pathways cross-interact rather than being single linear mechanisms. It has been suggested that cleavage of BID by caspase-8 can induce MOMP with subsequent release of cytochrome c into the cytoplasma [23].

WNV infection induces apoptosis in different cell types under involvement of components of the intrinsic and extrinsic apoptotic pathways. WNV infection as well as gene delivery of WNV capsid into the monkey kidney cell line Vero, induced apoptosis through a mitochondrial-based caspase-9 pathway [5,8]. In mouse neuroblastoma cell line Neuro-2a, WNV infection induced upregulation of bax gene expression [6]. Moreover, the WNV NS3 induced host cell apoptotic pathways involving caspase-8 and -3 in different cell types [7]. Recently, aspects of WNV-induced apoptosis in neural cells were studied in wild-type and caspase-3^-/- ^mice and primary CNS-derived mouse neurons [11]. Caspase-3 activation and DNA-fragmentation were demonstrated in brains of infected animals and in neuronal cultures. It was shown that, caspase-3 deficiency significantly decreased WNV-induced death of treated animals. Our results are in concert with these findings, demonstrating that our culture system may be useful to study virus-induced cell death in neuronal cells. In contrast to this recently published report [11], we also evaluated the apoptotic-pathways upstream of caspase-3 activation. Our data reveal that WNV infection induces caspase-8, -9 and -3 activation, PARP cleavage, MOMP, as indicated by cytochrome c release from the mitochondria, and cell death in T98G cells. The induction of apoptosis in T98G cells was replication-dependent as UV-inactivated virus did not induce death of treated cells. The WNV-induced PARP cleavage in T98G cells was strongly inhibited by the use of a pan-caspase inhibitor. Furthermore, inhibition of caspase activity inhibited virus-induced cell killing without affecting virus replication. This demonstrates that WNV-induced apoptosis plays a significant role in WNV-induced death of T98G cells.

WNV infection induced cleavage of the effector caspase-3 and the initiator caspases-8 and -9, suggesting contribution of both, the extrinsic and the intrinsic apoptotic pathways to WNV-induced apoptosis. In concordance, inhibition of caspase-8 as well as inhibition of caspase-9 significantly inhibited WNV-induced PARP cleavage. BID truncation/activation in WNV-infected T98G cells suggests cross-talk between the extrinsic and the intrinsic caspase pathways.

Inhibition of host cell apoptosis may improve outcome with or without affecting virus replication in different viral diseases. Two broad-spectrum caspase inhibitors were shown to inhibit reovirus-induced myocardial pathogenesis and to improve survival in mice, without exerting inhibitory effects on virus replication [24]. Coxsackievirus B3 induced apoptosis and cytopathic effects were prevented by inhibition of glycogen synthase kinase 3 beta (GSK3β) in cell culture [25]. Apoptosis inhibition also prevented cytopathic effects of flaviviruses. A pancaspase inhibitor prevented JEV apoptosis in murine (N18) and human (NT-2) neuronal cell lines [26]. Treatment of N18 cells with dehydroepiandrosterone suppressed JEV-induced cytopathic effects and JEV-induced apoptotic cell death without influencing virus replication [27]. Moreover, in vitro treatment of primary neurons with IFN-ß either before or after infection increased neuronal survival independently of its effects on WNV replication [28], demonstrating that neuroprotective agents might be useful to prevent WNV induced neural damage. However, cell death in vivo is much more complex and involves multiple regulators. To underscore a need for detailed studies of apoptosis it should be noted that there are recent studies showing that apoptotic cells may induce apoptosis in neighbouring healthy cells through bystander-mediated events [29,30]. This possibility has not been ruled out in the WNV infected brain.

## Conclusion

We found that WNV infection induces cell death in the brain-derived tumour cell line T98G by apoptosis under involvement of constituents of the extrinsic as well as the intrinsic apoptotic pathways. Pan-caspase inhibition prevented WNV-induced PARP cleavage as well as cell death without affecting virus replication. Our results illuminate the molecular mechanism of WNV-induced neural cell death.

## Methods

### Materials

Caspase inhibitor peptides z-lle-Glu(OMe)-Thr-Asp(OMe)-FMK (z-IETD-fmk), z-Leu-Glu(OMe)-His-Asp-(OMe)-FMK (z-LEHD-fmk) (Calbiochem, Darmstadt, Germany) and benzyloxycarbonyl-Val-Ala-Aspfluoromethylketon (z-VAD-fmk) (R&D Systems, Wiesbaden, Germany) directed against the active forms of caspase-8, caspase-9 and pan-caspase, respectively, were dissolved in dimethylsulfoxide (DMSO) at stock concentrations of 100 μM and stored at -20°C until use. They were added directly to culture medium at concentrations ranging from 6.25 to 100 μM. The concentration of DMSO did not exceed 0.1% (v/v) of total culture medium volume throughout all experiments.

### Cell cultures

T98G cells, derived from a patient with glioma and vero cells (monkey kidney cell line) were obtained from the American Type Culture Collection (ATCC, Rockville, MD). T98G cells were grown at 37°C in Iscove's modified Dulbecco's medium (IMDM) supplemented with 10% heat-inactivated fetal calf serum (FCS) and containing 100 IU/ml of penicillin and 100 μg/ml streptomycin. Vero cells were cultured in minimum essential medium (MEM) supplemented with 10%FCS and containing 100 IU/ml of penicillin and 100 μg/ml streptomycin.

### Virus preparation

WNV strain NY385-99, which was used throughout all experiments, was kindly provided by Dr. J. ter Meulen (Institut für Virologie, Phillips-Universität, Marburg, Germany). The strain was originally isolated from a snowy owl that died at the Bronx Zoo during the 1999 epizootic in New York City [13]. Virus was propagated on Vero cells and stocks were stored at -80°C. Virus titres were determined as 50% tissue culture infectious dose (TCID_50_/ml) in Vero cell monolayer in 96-well microtitre plates. For viral infection cells were incubated with virus at multiplicity of infection (MOI) 0.1 and 1.

WNV was inactivated by UV exposure (254 nm) for 20 min at a distance of 5 cm. Inactivation was confirmed by infection experiments on T98G and Vero cells.

In accordance with WHO recommendations, all work involving infectious WNV was performed under bio-safety level (BSL)-3 conditions.

### Cell viability assay

To assess effects of WNV infection on T98G cell viability confluent cell layers in 96-well microtitre plates were infected at MOI 0.1 and 1. The viability was measured at different time points post infection (p.i.) using the 3-(4,5-dimethyl-2-thiazolyl)-2,5-diphenyl-2H-tetrazolium bromide (MTT) assay method as described previously [14]. To assess the effects of z-VAD-fmk on cell viability of WNV-infected T98G cell cultures, the viability was measured using the trypan blue dye exclusion method. For this purpose, cells were washed twice with PBS and then trypsinised to gain a homogeneous cell suspension. Subsequently, 20 μl cell suspension was added to 80 μl of trypan blue vital staining dye. Following an incubation period of 2 min the amount of viable cells was determined by use of an inverted light microscope.

### Immune staining

Virus envelope-antigen, activated caspase-3 and the 85kD fragment of cleaved PARP were detected by immune-staining. The following primary antibodies were used: WNV-E1 (Chemicon, Hofheim Germany), caspase-3 (active) (R&D Systems, Wiesbaden, Germany) and PARP p85-fragment (Promega, Mannheim, Germany). Biotin-conjugated secondary monoclonal antibodies were used and visualisation was performed with streptavidin-peroxidase complex with AEC as a substrate.

### Western Blotting

Cell lysates were subjected to SDS-PAGE before transfer to nitrocellulose membranes (Schleicher & Schuell, Dassel, Germany) using the Mini-Protean II system (Bio-Rad, Munich, Germany). After transfer, blots were blocked in TBS containing 5% skimmed milk for 1 h to saturate the non-specific protein-binding sites on the nitrocellulose membrane. The following primary antibodies were used: WNV E1 (Chemicon, Hofheim, Germany), caspase-9 (kindly provided by Dr. Martin Zörnig, Frankfurt/Main, Germany), caspase-8 (Cell Signalling, Beverly, MA), BID (BioVision, Palo Alto, CA) and caspase-3 (Upstate, Charlottesville, VA). The blot was incubated with the primary antibody diluted in TBS overnight at 4°C with gentle agitation. Following a 1 h incubation period with peroxidase-conjugated secondary antibody at room temperature visualisation was performed by enhanced chemiluminescence using a commercially available kit (Amersham, Liverpool, UK).

### Cytochrome-c release

The cytochrome c release from mitochondria was detected by flow cytometry as previously described [15,16]. Briefly, mock infected and WNV infected (MOI 1) cells were harvested, washed with cold PBS, pelleted, and permeabilised so that cytochrome c, released from the mitochondria in apoptotic cells, could be washed away. Upon fixation with 3.7% formaldehyde, cells were incubated for 1 h at room temperature with blocking buffer (0.05% saponin, 3% BSA in PBS) followed by incubation overnight at 4°C with primary anti-cytochrome c antibody (BD Pharmingen, Heidelberg, Germany). After incubation with secondary FITC-labelled anti-mouse antibody for 40 min on ice, the percentage of non-apoptotic cells containing mitochondrial cytochrome c was quantified.

### Statistics

Results are mean ± S.D. of at least three independent experiments and images shown are representative for a set of three independent experiments. Student's t-test was performed to calculate significance with p values < 0.05 considered to be significant.

## Authors' contributions

MCK (first author) performed all the experiments, analyzed the data and drafted the manuscript. MM (second author) and HO participated in the design of the study, the interpretation of the data and performed the statistical analysis. HWD and JC conceived the study, coordinated and supervised the work, and helped in drafting the final manuscript. All authors have read and approved the manuscript.
